# Supplementary material for: Knowledge, attitudes and factors associated with the awareness of caregivers of under-five children regarding the malaria vaccine in the Tiko Health District, Cameroon: A community-based cross-sectional study
Source: PLOS Glob Public Health. 2026 Mar 4;6(3):e0004659. doi: 10.1371/journal.pgph.0004659 (PMC12959648; doi:10.1371/journal.pgph.0004659)
Supplement: Attachment — Submitted filename: REVIEW PLOS.docx [file pgph.0004659.s002.docx]

**Journal Reviewer Report:** Ahmad Suleiman Idris

**Date:** 18 May 2025

**Title:** Knowledge and attitudes of caregivers of under-five children toward the malaria vaccine in the Tiko Health District, Cameroon: A community-based cross-sectional study.

**Overall Impression**

This manuscript addresses a timely and significant public health issue: caregiver knowledge and attitudes towards the new RTS,S/AS01 malaria vaccine in a malaria-endemic region of Cameroon. The successful rollout of this vaccine, a crucial tool in combating malaria in under-five children, heavily relies on community acceptance, which is shaped by knowledge and attitudes. The study's focus on caregivers is appropriate, as they are the primary decision-makers for child health. The cross-sectional design is suitable for assessing the status of knowledge and attitudes. However, there are several areas that require clarification and strengthening before the manuscript can be considered for publication.

The background clearly sets the stage, highlighting the burden of malaria and the potential of the new vaccine. The rationale for the study is evident. The methods provide a good overview of the study design, period, location, sample size, sampling technique, data collection tool, and analysis software. However, as noted in major comments, more detail is needed for the scoring of knowledge (and attitude), questionnaire design (and standardization), sampling technique (etc.) in the full manuscript.

The results present key findings concisely. The discrepancy between awareness and actual good knowledge or positive attitudes is a striking and important finding. Strengths and limitations should be captured in the main article. The main article should have improved the conclusion and recommendations.

**Major Comments**

1. In text citations: line 60 “[1,2][3]” can be put in the same bracket as this [1-3]. Also, on line 67 “[8,8]” is a duplication of citation. Generally, for all in-text citations in the paper, I recommend the citations to be put at the end of the sentences for improved clarity. Example, in lines 67, 68, and 69; all the citations can be put at the end of the sentence like this [9-12].
2. Low knowledge and attitude levels: The results indicate that "only 26.6% demonstrated good knowledge, and 25.1% exhibited positive attitudes toward it" (lines 26-27). These figures are concerning and highlight a significant gap. The manuscript, particularly in the discussion section, needs to delve deeper into the reasons for these low levels. What specific misconceptions were prevalent beyond those related to "vaccine efficacy and purpose" (Line 29)? What are the potential drivers of negative attitudes?
3. Definition of "Good Knowledge" and "Positive Attitudes": The first thing is the assessment of the knowledge and attitude scores. What were the major domains these questions assessed. Was awareness of malaria vaccine part of the knowledge questions? More importantly, details on how the knowledge and attitude scores were constructed are needed. For example: How many questions were there for knowledge? What aspects of knowledge were assessed (e.g., schedule, side effects, efficacy, target age group, mechanism of action)? Similarly, the authors used a Likert scale for attitude, what domains or specific questions constituted the attitude score? Were these questions standardized during pre-testing? What was the Cronbach’s alpha?
4. Multistage Sampling Technique While "a multistage sampling technique" (Line 20) is mentioned in the Abstract: Methods, more detail is required in the full Methods section of the paper. This should include: How were the four health areas selected (randomly, as stated, but was there any stratification based on urban/rural or other characteristics)?How were households selected within these areas? How were caregivers then selected within these households (e.g., random selection if multiple eligible caregivers, specific criteria)? This detail is crucial for assessing the representativeness of the sample and the generalizability of the findings.
5. In table 1, the variable “ever attacked with malaria” choose a more appropriate word example ever “had”, ever been “infected” e.t.c. In table 2, after the variable “Doses of malaria vaccine” which variable have these responses: “At 1 year, at 3 months, at 6 months…”? and, after “malaria vaccine is 100% effective” which variable do these responses belong to: “Consult a healthcare provider…, Skip that dose…, Start the vaccination…, wait until the next…”?
6. In figure 1 (line 244), “side effects of malaria”. The title has incomplete information. Is this referring to side effects of the disease malaria itself or the vaccine? Please clarify. Also, figure 1 was not described in the paper. For figure 2 and 3, I suggest you remove them, create a table for knowledge and attitude scores or include them in your other tables, show the actual score categories. Figure 2 does not have a title.
7. In discussion, line 282, “some believing it treats malaria symptoms rather than prevents infection” was not captured by your results and has no citation if this was a finding by another study.
8. In discussion, lines 283 – 285, read the two studies again and confirm the information.
9. In discussion, lines 285—287, provide citation.
10. In discussion, lines 289 – 290, provide citation.
11. In discussion, line 291, “(Muriuki et al., 2021)” is a different referencing style, please use the Vancouver style for consistency.
12. In discussion, lines 291 – 293, provide citation.
13. Discussion fails to provide the strengths and limitations of the study.
14. The conclusion and recommendations are deficient; these could be improved.

**Minor Comments**

1. Role of healthcare providers: It's noted that "Healthcare providers were the most common source of information (35.4%)" (Line 28). The Discussion section of the full paper should explore how to leverage and strengthen this channel further. Were there differences in knowledge/attitude based on the source of information? This could be explored if such data were collected.
2. Statistical analysis: The authors focused on descriptive statistics, did they consider doing any inferential statistics (e.g., chi-square tests, t-tests, logistic regression) to explore association between sociodemographic characteristics and knowledge/attitude scores. This would add significant depth to the findings presented in the results section. Look at your reference 22. https://bmcpublichealth.biomedcentral.com/articles/10.1186/1471-2458-14-883/tables/2.
3. Pre-tested Questionnaire: It is good practice that the questionnaire was "pre-tested" (Line 21). The full methods section should briefly describe the pre-testing process (e.g., on whom it was pre-tested, sample size for pre-test, location of pre-test, and any significant modifications made to the questionnaire as a result). Where is the source of the questionnaire? Was the questionnaire adopted or adapted?
4. Instead of putting totals in your tables state the n for each variable eg. Sources of information (n=410)
