# Supplementary material for: Knowledge, attitudes and factors associated with the awareness of caregivers of under-five children regarding the malaria vaccine in the Tiko Health District, Cameroon: A community-based cross-sectional study
Source: PLOS Glob Public Health. 2026 Mar 4;6(3):e0004659. doi: 10.1371/journal.pgph.0004659 (PMC12959648; doi:10.1371/journal.pgph.0004659)
Supplement: Attachment — Submitted filename: Plos Global Public Health Review comments for authors.docx [file pgph.0004659.s003.docx]

**Abstract**

“*A descriptive cross-sectional survey*”. Please rephrase as “A cross-sectional survey”

**Background**

1. Check and correct references in line 60 and 67
2. “Moreover, caregivers' socio-economic 80 status, education level, and exposure to health education initiatives can further affect their understanding and acceptance of new interventions like the malaria vaccine”. Provide a reference
3. While the authors have demonstrated the importance of understanding caregivers knowledge and attitudes toward the malaria vaccine, the research gap that authors are seeking to close, is not clearly established. Has there been similar studies in the study context? Are there similar studies in other contexts in Cameroon or outside Cameroon but from the Sub-Saharan African region? What are the findings of those studies, and the research gap that need to be addressed. Overall, authors should consider delving into the literature of what has been done so far either within the study context or outside of the study context, and then highlight what new thin the current study is adding to the existing literature.
4. There is currently no theoretical underpinning for this study. A study like this must be rooted in in a relevant theoretical framework such as the theory of planned behaviours. While it is not compulsory to use the proposed theory, it is imperative to have the study guided by a relevant theory.

**Materials And Methods**

1. At line 90, please remove the word “descriptive”. Study design can be cross-sectional or longitudinal but not descriptive. Descriptive is part of the analytical approach.
2. “This study was conducted in some selected health areas of the Tiko Health District”. Please state the selected health areas.
3. The “ETHICAL CONSIDERATIONS” should follow immediately after the “Data Collection” section.

**Data Analysis**

- A sample size over 400 is large enough for test of association. While the authors did a good job with descriptives, that is too basic/not robust and cannot be used to confirm any hypothesis about knowledge and attitudes towards malaria vaccine. I strongly recommend a more advanced analysis/test of association for statistical significance. From the Tables, I can tell the variable “Aware of malaria vaccine”(outcome measure) is binary, hence a multivariable logistic regression model is more appropriate. See one the pioneering study on child malaria vaccine for guidance: <https://doi.org/10.1371/journal.pone.0296934>

**Results**

Kindly remove the “Totals” from Table 1 and 2.

**Discussion**

The revised version of the manuscript should discuss significant independent variables/predictors form the regression model(s), and discussions should be guided by the theoretical framework of the study as well.

Additional Comments: The study have merit. However, the methodology/analytical approach is currently weak and needs major revision for robustness.
