# Supplementary material for: Knowledge, attitudes and factors associated with the awareness of caregivers of under-five children regarding the malaria vaccine in the Tiko Health District, Cameroon: A community-based cross-sectional study
Source: PLOS Glob Public Health. 2026 Mar 4;6(3):e0004659. doi: 10.1371/journal.pgph.0004659 (PMC12959648; doi:10.1371/journal.pgph.0004659)
Supplement: Attachment — Submitted filename: RESPONSE TO REVIEWER COMMENTS_KAF1.docx [file pgph.0004659.s006.docx]

**RESPONSE TO REVIEWERS’ COMMENTS**

**Response to Reviewer 1 Comments**
We sincerely thank Reviewer 1 (Ahmad Suleiman Idris) for the thoughtful and detailed feedback, which helped improve the clarity and robustness of our manuscript. Below, we provide a point-by-point response, indicating how each comment has been addressed. All major changes are highlighted in **bold** in the revised manuscript.

**Major Comments**

**1. In-text citations formatting**

- **Response**: All in-text citations have been corrected. We removed duplicates (e.g., “[8,8]”) and unified the format using square brackets and the Vancouver style. Citations are now placed at the end of sentences, including lines 67–69, as recommended.

**2. Low knowledge and attitude levels**

- **Response**: We revised the **Discussion** section to more thoroughly examine possible reasons for the low levels of knowledge (26.6%) and positive attitudes (25.1%). Misconceptions identified include confusion about the vaccine schedule, uncertainty about effectiveness, and misunderstanding of eligibility age. We also discuss potential drivers of negative attitudes, such as misinformation, novelty of the vaccine, and lack of consistent health messaging.

**3. Definition of “Good Knowledge” and “Positive Attitudes”**

- **Response**: We have clarified how knowledge and attitude scores were constructed:
  - **Knowledge**: Seven (7) items with yes/no/don’t know responses. Each correct answer scored 1 point. A total of **≥60% (i.e., 4.2/7 ≈ 5 or more correct answers)** was considered good knowledge.
  - **Attitudes**: Seven (7) Likert-scale questions with options from **strongly disagree (1 point)** to **strongly agree (3 points)**. Total possible score was 21; participants scoring **≥60% (i.e., 13 or more points)** were categorized as having a positive attitude.
  - This explanation is now located in the **Results section** under *Overall Knowledge and Attitude*, and also briefly described in **Methods > Outcome Measures**.

**4. Multistage Sampling Technique**

- **Response**: The **Sampling Technique** section in the **Methods** has been revised to reflect that **six (not four)** health areas were selected. A **Probability Proportional to Size (PPS) sampling table** was added to show the distribution of participants across the six areas. The selection process within each area and household has been described in detail, including how eligible caregivers were randomly selected.

**5. Table 1 and Table 2 Variable Wording**

- **Response**:
  - “Ever attacked with malaria” has been corrected to **“Ever had malaria”**.
  - We clarified response categories in **Table 2**:
    - “At 1 year, 3 months, 6 months…” refers to the question: *“When should a child receive the malaria vaccine?”*
    - “Consult a healthcare provider… Skip that dose…” refers to: *“What action would you take if a dose is missed?”*

**6. Figures 1–3**

- **Response**:
  - **Figure 1** title was revised to: *“Reported side effects of the malaria vaccine among caregivers.”*
  - A brief description of Figure 1 is now included in the **Results**.
  - **Figures 2 and 3** were removed. A new **Table 3** now displays categorized knowledge and attitude scores for clarity.

**7. Discussion line 282: Incorrect statement**

- **Response**: The statement about caregivers believing the vaccine treats symptoms (not prevention) has been **removed**, as it was not supported by the findings or any citation.

**8–12. Discussion references (lines 283–293)**

- **Response**: All mentioned statements have been reviewed:
  - Interpretation errors were corrected.
  - **Missing citations have been added**, including for Muriuki et al.
  - APA-style citation formats were converted to **Vancouver style** to ensure consistency.

**13. Strengths and Limitations**

- **Response**: A new subsection was added at the end of the **Discussion**, clearly outlining:
  - **Strengths**: First study of its kind in the region, community-based design, adequate sample size, pre-tested tool.
  - **Limitations**: Cross-sectional design, self-reported data, limited use of inferential analysis, possible recall bias.

**14. Conclusion and Recommendations**

- **Response**: The **Conclusion** has been strengthened to summarize the implications of poor knowledge and attitudes. Recommendations now include:
  - Intensifying health education,
  - Using healthcare providers as primary messengers,
  - Tailoring messaging to address specific misconceptions.

**Minor Comments**

**1. Role of healthcare providers**

- **Response**: The **Discussion** has been expanded to reflect the important role of healthcare providers, who were the most cited source of information (35.4%). We suggest future interventions should focus on empowering providers with accurate, culturally sensitive messaging. Data on knowledge by source of information is now summarized in supplementary material.

**2. Statistical analysis**

- **Response**: As suggested, we have conducted **both simple and multiple logistic regression analysis to identify factors associated with awareness of the malaria vaccine**. These results are presented in the **Results** section. We note in the **Discussion** that future studies could benefit from multivariable models (e.g., logistic regression) to better adjust for confounders.

**3. Pre-tested Questionnaire**

- **Response**: The **Methods** section now includes:
  - The questionnaire was **adapted from WHO tools and prior literature** on vaccine acceptance.
  - It was pre-tested on **20 caregivers in Buea Health District** (not part of the study area).
  - Feedback was used to improve clarity and cultural relevance.

**4. Table formatting (Totals vs n)**

- **Response**: Done.

**Response to Reviewer 2 Comments**

We thank Reviewer 2 for the insightful and constructive feedback that has significantly strengthened the quality of our manuscript. Below is our detailed, point-by-point response to each of the comments.

**Abstract**

**Comment**: “A descriptive cross-sectional survey.” Please rephrase as “A cross-sectional survey.”

**Response**: Thank you. We have rephrased the sentence to:

**“A cross-sectional survey was conducted…”**
This correction has been made in the **Abstract** section.

**Background**

**1. Check and correct references in line 60 and 67.**

**Response**: We have reviewed and corrected the citation formatting and numbering in lines 60 and 67 to ensure accuracy and consistency with the Vancouver referencing style.

**2. “Moreover, caregivers’ socio-economic status…” – Provide a reference.**

**Response**: A supporting reference has been added:

**“Moreover, caregivers’ socio-economic status, education level, and exposure to health education initiatives can further affect their understanding and acceptance of new interventions like the malaria vaccine [13].”**

**3. The research gap is not clearly established. Expand literature review with studies from Cameroon/Sub-Saharan Africa.**

**Response**: We have substantially expanded the **Background** section to include:

- Existing literature from **Cameroon**, such as studies on malaria vaccine awareness in the Buea and Yaoundé health districts.
- Studies from other **Sub-Saharan African countries**, including Ghana, Nigeria, and Kenya, on caregiver knowledge and attitude toward the malaria vaccine.
- A clearer statement of the **research gap**, emphasizing that while some studies exist, **none have specifically focused on the Tiko Health District**, and few have examined both **knowledge and attitudes using a structured framework**.

**This study therefore fills a gap by assessing both knowledge and attitudes in a malaria-endemic and socio-culturally unique district, shortly after the vaccine's pilot introduction.**

**Materials and Methods**

**1. At line 90, remove “descriptive” from study design.**

**Response**: Corrected. The sentence now reads:

**“A community-based cross-sectional study was conducted…”**

**2. “This study was conducted in some selected health areas…” – Please specify.**

**Response**: We now specify that the study was conducted in **six health areas**: Holforth, Tiko Town, Likomba, Mutengene, Missellele, and Mudeka. This has been updated in the **Study Setting** subsection.

**3. Move “Ethical Considerations” to follow “Data Collection.”**

**Response**: Done. The **“Ethical Considerations”** section now immediately follows the **“Data Collection”** subsection for logical flow.

**Data Analysis**

**Comment**: The sample size (n > 400) is adequate for inferential analysis. The use of descriptive statistics alone is insufficient. Recommend multivariable logistic regression.

**Response**: Thank you for this important recommendation. We have revised our analytical approach:

- We conducted **bivariate analyses** using simple logistic regression to check association between socio-demographic variables and awareness of the malaria vaccine.
- We further fitted **multivariable logistic regression models** to identify independent predictors of:

These analyses are now presented in the **Results** section under “Factors associated with malaria vaccine awareness.” The corresponding **adjusted odds ratios (AORs), confidence intervals, and p-values** are reported in **Table 4 and Table 5**.

We referenced the study suggested by the reviewer for guidance:

**Results**

**Comment**: Kindly remove the “Totals” from Table 1 and 2.

**Response**: We have removed all instances of “Totals” in **Tables 1 and 2** and instead clearly stated the **sample size (n)** for each variable group, e.g., “Sources of information (n=410).”

**Discussion**

**Comment**: The revised manuscript should discuss significant independent variables/predictors and relate them to the theoretical framework.

**Response**: The **Discussion** section has been significantly expanded to:

- Highlight key predictors identified in the regression models (e.g., education level, source of information, health area).
- We have also compared our findings to similar studies within Cameroon and in the region, as previously requested in the expanded literature review.

**Additional Comments**

**Comment**: The study has merit but the methodology/analytical approach was initially weak.

**Response**: We agree and sincerely appreciate the reviewer’s guidance. The revised manuscript now presents a **much more robust methodology** and **analytical approach**, including:

- Multivariable inferential analysis,
- Improved discussion of implications based on findings.
