# Supplementary material for: Knowledge, attitudes and factors associated with the awareness of caregivers of under-five children regarding the malaria vaccine in the Tiko Health District, Cameroon: A community-based cross-sectional study
Source: PLOS Glob Public Health. 2026 Mar 4;6(3):e0004659. doi: 10.1371/journal.pgph.0004659 (PMC12959648; doi:10.1371/journal.pgph.0004659)
Supplement: Attachment — Submitted filename: Plos Global Public Health.docx [file pgph.0004659.s005.docx]

1. In the abstract, rephrase the following “Factors independently associated with caregivers’

awareness of the malaria vaccine were his of malaria in child (aOR:0.55, 95%CI:0.28-

0.97; p=0.40), trust in health workers (aOR:3.02, 95%CI:1.83-4.99, p<0.001), and

going for general children routine vaccination (aOR:3.57, 95%CI:2.27-5.60; p<0.001)” for clarity and standards in reporting. The current reporting lack clarity in terms of the direction of association with malaria vaccine awareness. Also, check the sentence for typos and grammatical error(s). Why do the authors report insignificant findings (aOR:0.55, 95%CI:0.28-0.97; p=0.40) ?. Clearly, the P-Value is not 0.05 and/or less. These corrections should be extended to the results section as well.

2. While authors used aOR in the reporting of results, they however used AOR on the tables. Authors should revise appropriately for uniformity. Also, all abbreviations should be stated in full at first appearance. Notably, abbreviations used on tables should be defined at the base of the table(s).
3. Authors should report on the goodness of model fit measures including the R-squared values and total number of observations. These should be included at the base of the Table(s) as in the attached screenshot example.

4. In my previous comments, I strongly stated the following “There is currently no theoretical underpinning for this study. A study like this must be rooted in in a relevant theoretical framework …….. While it is not compulsory to use the proposed theory, it is imperative to have the study guided by a relevant theory.” The authors however ignored this and stayed silent on that. I request for the second time that the study be underpinned by a relevant theoretical framework, and discussions should be guided by the theoretical framework of the study as well.
