# Supplementary material for: Knowledge, attitudes and factors associated with the awareness of caregivers of under-five children regarding the malaria vaccine in the Tiko Health District, Cameroon: A community-based cross-sectional study
Source: PLOS Glob Public Health. 2026 Mar 4;6(3):e0004659. doi: 10.1371/journal.pgph.0004659 (PMC12959648; doi:10.1371/journal.pgph.0004659)
Supplement: Attachment — Submitted filename: comments.docx [file pgph.0004659.s007.docx]

**Response to Reviewer Comments**

**Manuscript ID:** PGPH-D-25-01040R1
**Title:** Knowledge, attitudes and factors associated with the awareness of caregivers of under-five children regarding the malaria vaccine in the Tiko Health District, Cameroon

We sincerely thank the reviewer for the detailed and constructive comments. We have carefully addressed all concerns and revised the manuscript accordingly. All changes are highlighted in the revised manuscript. Our point-by-point responses are provided below.

**Reviewer Comment 1**

*In the abstract, rephrase the following… The current reporting lacks clarity in terms of the direction of association… Why do the authors report insignificant findings (p = 0.40)? These corrections should be extended to the results section as well.*

**Response**

We thank the reviewer for identifying this important issue. The p-value reported for history of malaria in the child was a typographical error. The correct p-value is **0.040**, which is consistent with the reported 95% confidence interval.

We have:

- Corrected the p-value in the **abstract, results text, and tables**
- Clearly stated the **direction of association** (less likely / more likely)
- Revised the sentence to meet standard epidemiological reporting conventions

The abstract and Results section have now been rewritten for clarity and accuracy.

**Reviewer Comment 2**

*Authors used aOR in the text and AOR in the tables. Revise for uniformity. All abbreviations should be stated in full at first appearance and defined at the base of tables.*

**Response**

We agree and appreciate this observation. We have:

- Standardized terminology to **Adjusted Odds Ratio (AOR)** throughout the manuscript
- Defined all abbreviations at first mention in the text
- Added abbreviation definitions at the base of all regression tables

This ensures consistency and improves readability.

**Reviewer Comment 3**

*Authors should report goodness-of-fit measures including R-squared values and total number of observations.*

**Response**

We thank the reviewer for this methodological recommendation. We have re-run the multivariable logistic regression analysis and now report the following model diagnostics:

- **Hosmer–Lemeshow goodness-of-fit test:** p = 0.670
- **Pseudo R²:** 0.299
- **Number of observations:** 410

These statistics are now reported in both the Results section and at the base of the multivariable regression table.

**Reviewer Comment 4 (Major)**

*There is currently no theoretical underpinning for this study… The study must be guided by a relevant theory and discussion aligned accordingly.*

**Response**

We sincerely apologize for not adequately addressing this important comment in the previous revision. In response, we have now explicitly grounded the study in the **Health Belief Model (HBM)**.

Specifically, we have:

- Added a new **Theoretical Framework** subsection in the Background (Line 99-109)
- Explicitly linked key study variables (knowledge, attitudes, trust in health workers, prior malaria experience, routine immunization) to HBM constructs
- Revised the **Discussion section** to interpret findings through the HBM, including perceived benefits, barriers, cues to action, and self-efficacy

We believe this theoretical integration substantially strengthens the conceptual rigor and interpretability of the study.

**Closing Statement**

We are grateful to the reviewer for the insightful comments, which have significantly improved the clarity, methodological rigor, and theoretical grounding of our manuscript. We hope that the revisions satisfactorily address all concerns and that the manuscript is now suitable for publication.
