# Supplementary material for: Knowledge, attitudes and factors associated with the awareness of caregivers of under-five children regarding the malaria vaccine in the Tiko Health District, Cameroon: A community-based cross-sectional study
Source: PLOS Glob Public Health. 2026 Mar 4;6(3):e0004659. doi: 10.1371/journal.pgph.0004659 (PMC12959648; doi:10.1371/journal.pgph.0004659)
Supplement: Attachment — Submitted filename: RESPONSE TO REVIEWERS F.docx [file pgph.0004659.s009.docx]

**POINT-BY-POINT RESPONSE TO REVIEWER COMMENTS**

We thank the reviewer for the constructive and insightful comments that helped us strengthen the quality, clarity, and rigor of our manuscript. Below, we provide a detailed, point-by-point response. All corresponding changes have been incorporated into the revised manuscript.

**ABSTRACT**

**Comment 1:**

*L16-17 – “The introduction of the RTS,S/AS01 (Mosquirix) malaria vaccine offers hope,” you should also mention R21…*

**Response:**
Thank you for this important suggestion. We revised the introductory sentence in the abstract to reflect both major malaria vaccines (RTS,S/AS01 and R21), acknowledging that R21 has not yet been introduced in Cameroon.
**Revision made.**

**Comment 2:**

*L24 – No need to mention SPSS version in the abstract.*

**Response:**
We removed the reference to “SPSS version 26” from the abstract to improve clarity and reduce word count.

**Comment 3:**

*L26 – What is the scoring system?*

**Response:**
We added a brief description of the scoring system used to classify knowledge and attitudes, specifying how scores were computed and how the threshold was applied.

**Comment 4:**

*L30 – Age should be summarized as median (IQR) instead of mean (SD).*

**Response:**
We recalculated age, summarized it as **median and interquartile range**, and updated the abstract accordingly.

**Comment 5:**

*No need to include p-values in the abstract when 95% CI is reported.*

**Response:**
We removed all p-values from the abstract and maintained only AORs with 95% CIs.

**Comment 6:**

*L39-41 – The section repeats earlier results; this should be a conclusion.*
We replaced the repeated results with a concise, focused conclusion summarizing the main implication of the study.

**MATERIALS AND METHODS**

**Comment 7:**

*L126-127 – Date information was repeated.*

**Response:**
The repeated date information was removed.

**Comment 8:**

*L133-134 – “What is the proportion?” Clarify.*

**Response:**
We clarified that the proportion refers to the **estimated proportion of caregivers aware of the malaria vaccine**, which informed the sample size.

**Comment 9:**

*L140 – Explain why sample size was made up to 410.*

**Response:**
We added an explanation stating that the sample size was increased from 384 to 410 to improve precision and to account for more representativeness across sampled health areas.

**Comment 10:**

*L178 – You wrote four sections but presented only three sections.*

**Response:**
We corrected this inconsistency by updating the text to reflect the actual three sections.

**Comment 11:**

*L238 – SPSS should be spelled out when first mentioned.*
We spelled out **Statistical Package for the Social Sciences (SPSS)** when first introduced.

**Comment 12:**

*L242 – No pie charts; not needed.*
We removed reference to pie charts and retained only tables for categorical variables.

**Comment 13:**

*L248 – Explain why the cutoff of 60% was used.*

**Response:**
We added a justification explaining that the 60% cutoff was based on methods used in similar KAP studies in Africa, where ≥60% reflects “adequate” knowledge/attitude levels.

**RESULTS**

**Comment 14:**

*L258 – “under children” is a typo.*

**Response:**
Typographical error corrected.

**Comment 15:**

*Table 1 – Correct categories (>4 instead of 4+; >100,000 instead of 100000+). Specify currency and convert to USD.*

**Response:**
We corrected the category labels and added the currency (XAF), including approximate USD equivalents using the average exchange rate.

**Comment 16:**

*Table 2 – Consider age eligibility for vaccine doses.*

**Response:**
We added a footnote clarifying that full vaccination requires four doses, but children under five may not yet be eligible for the later doses depending on their age at first immunization.

**Comment 17:**

*Figure 1 unnecessary—could be a table.*

**Response:**
Thank you for this suggestion. We fully understand the reviewer’s concern regarding the necessity of Figure 1. However, after careful consideration, we believe that retaining the figure adds important value for the following reasons:

The variable displayed (perceived side effects) includes multiple categories, and a bar chart allows readers to easily compare relative frequencies at a glance, which is more difficult to appreciate from a table.

The figure highlights a key public health finding—that “I do not know” is the most frequent response (31.8%), visually emphasizing the high level of uncertainty and misinformation among caregivers. This visual emphasis supports one of the main discussion points regarding knowledge gaps.

PLOS journals encourage the use of simple figures to enhance data interpretation when they help readers quickly understand patterns, especially in descriptive results.

A table version would only repeat the information that is already presented textually in the Results section, while the figure provides a clearer and more intuitive summary without increasing word count.

Nevertheless, we revised the figure for clarity and ensured that it adheres to PLOS formatting standards (clean axis labels, readable percentages, and simplified color scheme).

We therefore respectfully request to retain Figure 1, as it enhances understanding of the distribution of perceived vaccine side effects.

**Comment 18:**

*L279-281 – Repetition about scoring system.*

**Response:**
The repeated sentences were removed.

**DISCUSSION**

**Comment 19:**

*L338–339 – Not clear how guided by the HBM; include model details in Methods.*

**Response:**
We added a paragraph in the Methods under **“Conceptual Framework”** explicitly describing the Health Belief Model and how it guided variable selection and interpretation.
We also expanded the discussion to link findings more concretely to HBM constructs (perceived susceptibility, perceived barriers, cues to action).

**Comment 20:**

*Difference between 30% and 40% may not be meaningful without CIs.*

**Response:**
We revised the discussion to acknowledge that the comparison should be interpreted with caution and added text emphasizing that differences without confidence intervals cannot be interpreted as statistically significant.

**Comment 21:**

*Sample size may not be enough for logistic regression; mention as limitation.*

**Response:**
Although the sample size is adequate for prevalence estimation, we agreed that the events-per-variable ratio for multivariable logistic regression may be limited.
We added this as a study limitation, explaining possible sparse data effects and wide CIs for some predictors.

**CLOSING STATEMENT**

We are grateful for the reviewer’s thorough and insightful feedback. The manuscript has been substantially strengthened through these revisions. We believe the updated version is now clearer, more rigorous, and more aligned with the journal’s expectations.
