# Supplementary material for: Knowledge, attitudes and factors associated with the awareness of caregivers of under-five children regarding the malaria vaccine in the Tiko Health District, Cameroon: A community-based cross-sectional study
Source: PLOS Glob Public Health. 2026 Mar 4;6(3):e0004659. doi: 10.1371/journal.pgph.0004659 (PMC12959648; doi:10.1371/journal.pgph.0004659)
Supplement: Attachment — Submitted filename: Response to editor.docx [file pgph.0004659.s010.docx]

Below is a **ready-to-submit response to the editor**.
You can copy and paste this directly into your **“Response to Reviewers”** file.

**Response to the Academic Editor**

Manuscript ID: **PGPH-D-25-01040R3**
Title: Knowledge, attitudes and factors associated with the awareness of caregivers of under-five children regarding the malaria vaccine in the Tiko Health District, Cameroon: A community-based cross-sectional study

Dear Dr. Ashton,

We sincerely thank you for your careful editorial review of our manuscript and for your constructive suggestion to further enhance the interpretation and contextualisation of our findings.

**Editor’s comment:**
Please consider including a sentence or two in the introduction section explaining when RTS,S was approved for use in Cameroon, and when the vaccine was first available to children within the study district.

**Response:**
We have revised the Introduction section to include a brief paragraph specifying the timing of the approval and rollout of the RTS,S/AS01 malaria vaccine in Cameroon, as well as the period when the vaccine became available to under-five children in the Tiko Health District. This information was added immediately after the paragraph describing the WHO recommendation and efficacy of the RTS,S/AS01 vaccine.

The added text clarifies that the malaria vaccine was approved for use in Cameroon following the WHO recommendation in 2023 and that routine rollout occurred subsequently, with availability to eligible children in the Tiko Health District in 2024, prior to the conduct of this study. We also explicitly state that vaccine introduction was still at an early phase during the study period, to aid interpretation and comparison with other settings as scale-up continues.

This revision has been referenced using authoritative sources from the World Health Organization.

We believe this addition improves the contextual understanding of our results and addresses the editorial recommendation.

We thank you again for your valuable guidance.

Sincerely,
The Authors
